# The assessment of microencapsulated *Lactobacillus plantarum* survivability in rose petal jam and the changes in physicochemical, textural and sensorial characteristics of the product during storage

**DOI:** 10.1038/s41598-022-10224-w

**Published:** 2022-04-13

**Authors:** Fateme Shoaei, Ali Heshmati, Reza Mahjub, Amir Daraei Garmakhany, Mehdi Taheri

**Affiliations:** 1 Department of Nutrition and Food Safety, School of Medicine, Nutrition Health Research Center, Hamadan University of Medical Sciences, Hamadan, Iran; 2 Department of Pharmacology and Toxicology, School of Pharmacy, Medicinal Plants and Natural Products Research Center, Hamadan University of Medical Sciences, Hamadan, Iran; 3 Department of Food Science and Technology Toyserkan, Faculty of Industrial Engineering, Bu-Ali Sina University, Beheshti Ave, Bahri Esfahani Ave, Toyserkan City, Hamadan, Iran

**Keywords:** Biological techniques, Microbiology

## Abstract

The present study aimed to develop a probiotic rose petal jam containing microencapsulated *L. plantarum.* The attributes of *L. plantarum* microcapsules and bacteria viability in simulated gastrointestinal conditions and jam were assessed. In addition, *L. plantarum* effects on physicochemical, textural and sensorial properties of jam were studied. The microencapsulation yield, diameter, and zeta potential value of the microcapsules ranged from 90.23 to 92.75%, 14.80–35.02 µm, and − 16.83 to − 14.71 mV, respectively. The microencapsulation process significantly increases the survival of *L. plantarum* in simulated gastrointestinal tract and jam. In jam samples containing *L. plantarum* microencapsulated with 2% sodium alginate and 3.5% or 5% Arabic gum and stored for 90 days, the bacterial count was higher than the acceptable level (10^6^ CFU/g). While there was no significant difference (*P* > 0.05) between physicochemical characteristics of non-probiotic and probiotic jams, taste and overall acceptance scores of microencapsulated probiotic jams were higher. The microencapsulation of *L. plantarum* in sodium alginate (2%) and Arabic gum (5%) and its inoculation into rose petal jam could yield a new probiotic product with increased health benefits.

## Introduction

Jam preparation has been one of the most common and low-cost methods for preserving fruits and supplying them in the off season^1^. According to Iranian standards, jam is defined as an intermediate-moisture product with a total soluble solids (TSS) content of 65°Brix (depending on the type of fruit used) of at least 20–35%^1,2^. Jams are a good source of energy, carbohydrates, and fiber and contain low levels of fat. They can be produced from various types of fruits. Rose petal jam is one of the highest consumed jams in Iran and in some other countries, including India^3^.

*Rosa damascena* Mill., known as Damask rose, belongs to the *Rosaceae* family^4^. In Iran, Damask rose is known as Gole Mohammadi^5^. It is utilized in the perfume, medicine, and food industries. Jam is the most important products prepared using Damask rose^3,5^. Rose petal jam is used as a tonic and laxative. It is a good source of powerful antioxidants and has various health benefits, e.g., it helps alleviate dysmenorrhea and fluid retention^6^.

At present, due to consumer awareness regarding the benefits of functional foods, the demand for the production and supply of these products is increasing^7^. Probiotics are one of the most important functional foods; they are live microorganisms that have beneficial effects on host health when consumed in sufficient amounts^8,9^. The number of viable probiotic cells at the time of consumption should be more than 10^6^ colony-forming unit (CFU)/g to exert a health benefit to humans^10^. Probiotics reduce the risks conferred by mutagens and carcinogens, improve lactose tolerance and the intestinal flora, and strengthen the immune system^11,12^. Most probiotics used in foods belong to the genera *Lactobacillus* and *Bifidobacterium*^13^.

Probiotics are mainly added to dairy products. However, given the presence of cholesterol in milk and the lactose intolerance and sensitivity of some people to dairy products, non-dairy probiotic products, such as cereal, legume, fruit, and vegetable based foods and drinks, have been developed^14,15^. Therefore, the addition of probiotics to plant foods can be a suitable alternative for the consumption of these bacteria in vegan diet and people who cannot consume dairy products^14,16^. Plant-based probiotics are cheaper sources and contain various phytochemicals^16^.

When probiotics are inoculated into a new foodstuff or drink, it is important to ensure that they preserve their viability during storage. The viability of probiotics depends on the oxygen level, final acidity of the product, water activity (AW), storage temperature, and presence of antimicrobial compounds^13,17^. Therefore, microencapsulation is an important method for increasing the survival of probiotics in foods during storage and transfer through the gastrointestinal tract^18^ From a microbiological point of view, microencapsulation is the coating of probiotics with various substances, including gums, maltodextrin, starch, chitosan, and gelatin, etc. to protect them from the external environment^19^.

There are few studies about probiotic jam production. Only one study investigated the viability of probiotic bacteria inoculated into peach jam^10^ and there is no published information about the production of probiotic rose petal jam. The viability of probiotics in jam could be reduced during storage because the low AW of the product and storage conditions (mostly at room temperature) could result in the death of probiotic bacteria. The aim of the present study was to assess the survival of free/microencapsulated *L. plantarum* in simulated gastrointestinal conditions and jam. In addition, probiotic effects on the physicochemical, textural, and sensorial characteristics of jam during storage at different temperatures were investigated.

## Materials and methods

### Materials

Sodium alginate and Arabic gum were obtained from Sigma Aldrich (St-Louis, USA). MRS agar and MRS broth were produced by Liofilchem (Roseto degli Abruzzi, Italy). Sodium hydroxide (NaOH), calcium chloride (CaCl_2_), potassium monophosphate (H_2_PO_4_), hydrochloric acid (HCl) and sodium citrate, pepsin and pancreatin and other chemicals were also purchased from Merck (Darmstadt, Germany). Lyophilized *L. plantarum* (ATCC 8014, PTCC: 1058), was bought from the Persian Type culture collection (Tehran, Iran).

### Preparation of viable cells and suspension of *L. plantarum*

Lyophilized *L. plantarum* was reactivated twice in MRS broth at 37 °C for 24 h. The cell suspension was centrifuged (ROTOFIX 32A, Hettich, Germany) at 4000 × g for 6 min at 4 °C. After the removal of the supernatant, 0.85% sterile normal saline solution was added and mixed to obtain free *L. plantarum* suspension^18,20^. The bacterial number in this solution that was counted according to the previous study^13^ was equal to 6 × 10^10^ CFU/mL.

### Microencapsulation of *L. plantarum*

The microencapsulation of *L. plantarum* by sodium alginate and Arabic gum was performed according to previous studies with some modifications^21,22^. First, a sodium alginate solution (1, 1.5 and 2% w/v) was prepared and sterilized (121 °C for 15 min). Two mL of *L. plantarum* suspension (6 × 10^10^ CFU/mL) was inoculated into 98 mL of sodium alginate solution and blended gently using a magnetic stirrer^21^. The obtained suspension was then dropped into sterile calcium chloride (1 L, 2% w/v) from a height of 20 cm through a sterile disposable needle with a diameter of 0.4 mm. After 20 min, beads were acquired by filtration through Whatman filter paper (No. 4) and washed with sterile deionized water^21^.

For second-layer formation on beads, Arabic gum was utilized. Arabic gum (2, 3.5 and 5% w/v) was dispersed in hot distilled water (75 °C) and stirred (600 RPM, 30 min) until complete dissolution. Afterwards, the beads were immersed into the sterilized Arabic gum (100 mL) and stirred for 30 min at 600 RPM^18^. Then they were transferred into the sterile glass, pre-frozen (− 20 °C, 2.5 h), placed in the freezer (− 70 °C, 12 h) for complete freezing, and dried in vacuum-freeze dryer equipment (operon, Hwanggeum, Korea) at − 55 °C for 24 h^20^. The obtained beads or microcapsules were collected in polyethylene bags and stored at 4°C^20^.

### Microencapsulation efficiency

The efficiency of microencapsulation was determined according to the method suggested by Arepally and Goswami, 2019 and Chen et al*.* 2017.

1 $$ {\text{Microencapsulation efficiency }} = \frac{{\left( {N \times M} \right)}}{{\left( {N0 \times V} \right)}} \times 100 $$

where N is *L. plantarum* count (log CFU/g) in microcapsules, M is the mass of the obtained microcapsule, N_0_ is *L. plantarum* count (log CFU/g) in cell suspension, V is the volume of *L. plantarum* suspension used for microencapsulation preparation^18,23^.

For *L. plantarum* count (log CFU/g) in microcapsules, 1 g of dried powder of the microencapsulated *L. plantarum* was suspended in 99 mL of sodium citrate (2%) solution^21^. The suspension was homogenized (100 RPM, 20 min, 37 ^○^C) by a shaker (Teifazma, Tehran, Iran). After the preparation of appropriate dilutions (tenfold serial dilutions in 0.85% saline solutions), one mL of each dilution was plated on MRS agar. *L. planetarium* was counted after incubation in anaerobic jar (Gas Pack, Anaerogen TM, Oxoid) at 37 °C for 24 h. Plates containing 30–300 colonies were counted, and *L. plantarum* number for each was recorded and reported as log CFU/g of dried powder^20^.

### The measurement of microcapsules size

The average size of the microcapsules was determined by a laser light diffraction Malvern (Malvern Instruments, Malvern, Worcestershire, UK) instruments^24^.

### The measurement of zeta potential

The zeta potential of microcapsules diluted to 0.001% with deionized water was measured^25^ with zetasizer particle size analyzer (Malvern Instruments Ltd. Malvern, Worcestershire, UK).

### Morphology of microcapsules

The morphology of microcapsule was measured with a scanning electron microscopy (SEM) (JEOL Co., Akishima, Japan) according to procedure suggested by previous study^25^.

### Measurement of moisture and AW of microcapsules

The moisture content of microcapsules was determined gravimetrically in oven (Shimaz Inc., Tehran, Iran) at 105 °C for 24 h. AW was measured at 25 ± 1 °C using a water activity meter (LabMaster. aw, Novasina, Switzerland). Before AW measurement of microcapsules, the equipment was calibrated with distillate water for result accuracy^18^.

### The preparation of simulated gastric and intestinal juice

The simulated gastric juice (SGJ) was prepared according to the method suggested by Annan et al.^26^ Therefore, pepsin (0.3% w/v) was added into sterile sodium chloride solution (0.2% w/v), and its pH was adjusted to approximately 2 with HCl (0.1 N) and filtrated through a membrane (0.45 µm, Millipore, Spain) for sterilizing.

For simulated intestinal juice (SIJ) preparation, pancreatic (0.1% w/v) and bile salts (0.45% w/v) were added into citrate sodium (2%). The mixture’s pH was adjusted to 7.4 with NaOH (0.1 N) and sterilized by filtration through a membrane (0.45 µm Millipore, Spain). Both gastric and intestinal juices were prepared and used daily^26^.

### Survival assay of free and microencapsulated *L. Plantarum* after sequential incubation in simulated gastrointestinal conditions

To assess the viability of *L. plantarum* under a simulated gastrointestinal condition (SGC), the applied method was similar to that used by Fareez et al.^28^ and Krasaekoopt and Watcharapoka^27^, with some modifications. Free *L. plantarum* suspension (1 mL) or microencapsulated *L. plantarum* (1 g) was added into falcon tubes containing SGJ (9 mL) and placed into a shaker incubator (100 RPM, 37 °C) for different times (0, 30, 60, 90, 120 and 180 min)^28^. After neutralizing with NaOH solution (0.1 N), the treated *L. plantarum* was removed from the SGJ by centrifugation (4,000 g, 10 min). The beads and cells were washed once with sterile distilled water and transferred into in a falcon tube containing SIJ (10 mL). The tubes were placed in a shaker-incubator at 37 °C for 180 min. Furthermore, beads and cells were removed by centrifugation (4000 g, 10 min), dissolved in sodium citrate (2%), and cultured as explained in the above Section^20^.

### The preparation of probiotic rose petal jam

**Figure 1:**
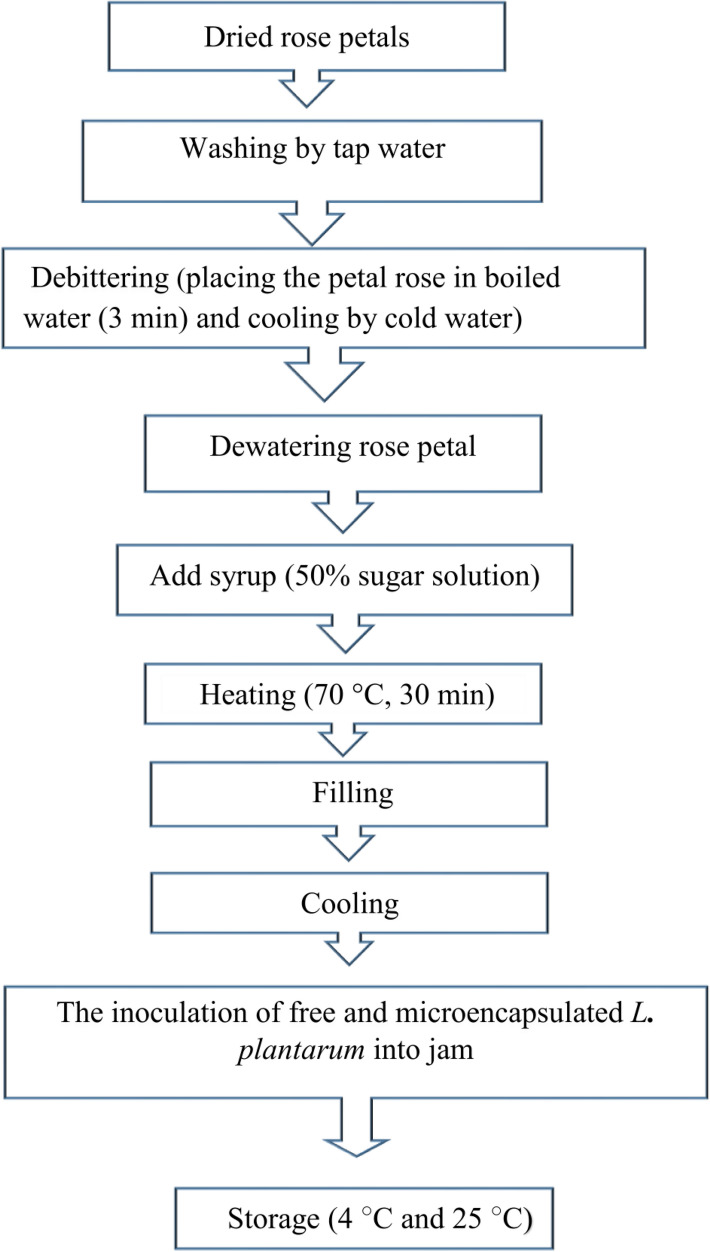
The diagram of probiotic rose petal jam production.

A schematic diagram of the production stages of the rose petal jam is provided in Fig. 1. In summary, 50 g of dried rose petals were placed in boiling water for 3 min to remove the bitter taste. Then, the samples were placed in cold water for 5 min. In the final step, the samples were poured over a sieve to drain out the water. Debittered rose petal jam samples were placed in a stainless steel pot. Syrup (a 50% sugar solution) was added and heated for 30 min. Finally, 1 tablespoon of lemon juice was added. When the samples’ temperature reached 45 °C, they were poured into glass containers. One mL of free *L. plantarum* suspension (6 × 10^10^ CFU/mL) or one g of micencapsulated *L. plantarum* (approximately 1.2 × 10^10^ CFU/g) was added into 100 g of jam. The samples were kept at 2 temperature conditions (4 and 25 ^○^C) for 90 days^10,29^. Because *L. plantarum* microencapsulated in sodium alginate (1%) and Arabic gum (2, 3.5 and 5%) had low survival under a simulated gastrointestinal condition, they were not inoculated in the jam. The photos of probiotic and non-probiotic rose petal jam were shown in Supplementary Fig. S1.

### Enumeration of *L. Plantarum in jam*

One g of jam samples stored for 1, 15, 30, 45, 75, and 90 days at room or refrigerator temperature was added into a falcon tube containing 9 mL peptone water and stirred. After preparing of serial (tenfold) dilutions, the number of bacteria was counted by a pour plate method using MRS agar. Plates were placed in an anaerobic jar and incubated at 37 °C for 24 h^29^.

### The assessment of physicochemical, textural and sensorial characteristics of probiotic rose petal jam

#### Determination of TSS

The TSS value of rose petal jam samples was measured by the digital refractometer (Krüss Optronic GmbH, Hamburg, Germany) at ambient temperature (20 °C) and expressed in °Brix^2^.

#### The measurement of titratable acidity

The acidity of rose petal jam specimens was determined by titrating with sodium hydroxide solution (0.1 N) in the presence of phenolphthalein indicator and expressed as citric acid percentage^2^.

The pH was directly determined by pH meter (Century, Model CP931, Bangalore, India).

#### The measurement of total non-reducing sugars

Total non-reducing sugar percentage of the rose petal jam was determined by the Lane and Eynon method^30^.

#### The color evaluation

A color measurement was performed similar to that carried out in our previous study^31^. Rose jam samples were placed on the floor of an aluminum dark chamber with 30 × 40 × 40 cm dimensions. Four 60-W halogen lamps were in the corners of the chamber. Photos were taken with a camera (Canon, Kanagawa, Japan). The color parameters including L* (brightness), a* (red-green), b* (yellow-blue) of the jam were determined using Photoshop 7.0 software.

#### The evaluation of texture and viscosity

Viscosity and texture properties including firmness, cohesiveness and chewiness were measured using a texture analyzer (Zwick, Ulm, Germany). Texture analysis was carried out using a back extrusion test in 2 cycles. Jam samples were poured into plastic containers 50 mm in height. A cylindrical probe (20 mm in diameter) was applied to compress the sample to a 25 mm depth with a speed of 40 mm/min^32^.

#### The sensory evaluation

The sensory properties (taste, odor, appearance, and overall acceptability) of the rose petal jam were evaluated according to the hedonic scale of nine points (from 9 = like extremely to 1 = dislike extremely). Trained panelists (15 men and 15 women aged from 18 to 45 years old) were used for sensory testing. The samples were kept at 20 °C temperature for 3 h and then examined for sensory properties^33^. A number code was considered for each sample. Then, the samples were poured into a sterile plastic spoon and given to panelist with their code randomized order. The panelists rinsed their mouths after testing each sample. The mean sensory scores were used in the analysis.

### Ethical approval

The protocol of the sensory experiments was approved by the Ethics Committee of Hamadan University of Medical Sciences (ethics code: IR.UMSHA.REC.1397.1030). Informed consent was obtained from human panelists for sensory evaluation. All methods were performed in accordance with the relevant guidelines and regulations.

### Statistical analysis

All the experiments were carried out in triplicate and their mean ± standard deviation were reported. The data were subjected to a one-way ANOVA and Duncan’s multiple range test to determine significant difference at the level of 95% using SPSS 22:0 Advanced Statistics (IBM; Armonk, NY, USA).

## Results and discussion

### Physicochemical properties of the microcapsules of *L. plantarum*

#### Microencapsulation efficiency

In this study, we used various levels of sodium alginate and Arabic gum for microencapsulation of *L. plantarum.* The statistical results showed the significant differences (*P* ≤ 0.05) in the efficiency of used various concentration levels (Table 1) so that the increased efficiency values was obtained at higher concentrations of sodium alginate (1.5 and 2%) and Arabic gum (2, 3.5 and 5%). Microencapsulation efficiency value ranged from 90.97 ± 4.68 to 98.86 ± 2.97%. In the previous study done by De Almeida Paula et al.^25^ the microencapsulation efficiency of *L. plantarum* coated by Arabic gum and gelatin was reported as 97.8% that it was similar to our findings. The increment of sodium alginate and Arabic gum concentration lead to formation of microcapsules with the thicker layer and a higher probiotic count. On the other hand, a thicker coating could protect the probiotic viability during freeze-drying better than a thin coating^34^. The high microencapsulation efficiency indicated that sodium alginate and Arabic gum were compatible matrices for microencapsulation of probiotics such as *L. plantarum.*

**Table 1:** Physicochemical properties of the *L. plantarum* microcapsules.

Parameter	Al_1_Ar_2_	Al_1_Ar_3.5_	Al_1_Ar_5_	Al_1.5_Ar_2_	Al_1.5_Ar_3.5_	Al_1.5_Ar_5_	Al_2_Ar_2_	Al_2_Ar_3.5_	Al_2_Ar_5_
Encapsulation efficiency (%)	90.97 ± 4.68^c^	91.75 ± 3.73^c^	92.19 ± 5.6^c^	92.3 ± 2.66^bc^	93.42 ± 6.85^b^	94.64 ± 4.97^b^	95.23 ± 5.93^ab^	97.97 ± 3.78^a^	98.86 ± 2.97^a^
Diameter (µm)	14.80 ± 1.23^c^	19.30 ± 2.90^bc^	21.71 ± 3.84^b^	16.12 ± 1.42^c^	21.26 ± 1.43^b^	31.12 ± 3.56^a^	16.92 ± 2.14^c^	24.50 ± 3.21^b^	35.02 ± 2.18^a^
Zp (-mV)	15.81 ± 0.30^a^	16.42 ± 0.36^a^	16.30 ± 1.32^a^	14.71 ± 0.76^a^	16.83 ± 1.7^a^	16.40 ± 0.95^a^	15.82 ± 0.53^a^	16.20 ± 2.27^a^	15.77 ± 0.87^a^
Moisture (%)	8.56 ± 2.22^a^	7.70 ± 1.13^a^	5.9 ± 2.06^b^	7.8 ± 1.75^a^	6.60 ± 1.18^ab^	5.50 ± 3.08^b^	7.87 ± 0.23^a^	5.67 ± 1.53^b^	4.38 ± 3.5^b^
AW	0.18 ± 0.01^a^	0.18 ± 0.01^a^	0.16 ± 0.02^a^	0.15 ± 0.00^ab^	0.13 ± 0.02^b^	0.12 ± 0.03^b^	0.15 ± 0.04^ab^	0.14 ± 0.39^ab^	0.11 ± 0.05^b^

Values are expressed as mean ± standard deviation (*n* = 3). Means followed by different lowercase letters differ statistically in the same column (*P* ≤ 0.05). AlAr shows *L. plantarum* microencapsulated with levels of 1, 1.5 and 2% sodium alginate (Al) and 2, 3.5 and 5% Arabic gum (Ar).

Encapsulation efficiency impacts the final number of probiotics in food and their viability in the gastrointestinal tract^22,35^. A previous study by Arepally and Goswami (2019) showed that the increasing Arabic gum concentration resulted in increased encapsulation efficiency, that it was similar to our results^18^.

#### Microcapsule size and morphology

The diameter of the prepared microcapsules in the current study ranged from 14.80 ± 1.23 to 35.02 ± 2.18 µm and depended on the sodium alginate and Arabic gum concentration (Table 1). Microcapsules more than 1000 µm in diameter had a negative impact on the sensory attributes of products. On the other hand, microcapsules with a size of less than 1 µm were unstable^36^. Microcapsule size may affect different properties such as solubility, water activity, and the number of viable probiotic cells^37^. The size of prepared *L. plantarum* microcapsules in the present study was small enough to avoid any negative effects on sensorial quality. According to the previous study, microcapsule size was directly proportional to hydrogel material viscosity: the higher viscosity lead to the larger particles size^38,39^. Therefore, it seemed that increasing sodium alginate and Arabic gum concentrations resulted in enhanced viscosity of prepared solutions, which could cause an increase in microcapsule size.

Supplementary Figs. S2 and S3 depicts the morphology (at magnifications 50 × and 5000 ×) of *L. plantarum* microencapsulated with different levels of sodium alginate and Arabic gum. Due to the ionic bond between the carboxyl group of sodium alginate and calcium, the structure of the microcapsules became spherical. Sodium alginate was also placed as a first layer on a suitable substrate of the second layer. Increased concentrations of sodium alginate and Arabic gum led to a smoother microcapsule surface. Therefore, the microcapsules formed with 2% sodium alginate and 5% Arabic gum had the smoothest surface (Supplementary Fig. S2). A smooth surface increases the protective properties of microcapsules and increases probiotic cell survival (Kalita et al*.* 2018). Previous studies have found that the morphology of microcapsules depends on the type of coating and its concentration^40^.

#### Zeta potential of microcapsules

As it can be seen in Table 1, the zeta potential value did not differ significantly (*P* > 0.05) among the different treatments. The zeta potential value ranged − 16.83 to − 14.71 mV. Sodium alginate and Arabic gum could be uniformly layered. The occurrence of carboxylic group of sodium alginate and Arabic gum created negative zeta potential^34^ Lee and Chang (2020) found that high zeta potential values prevent aggregation of emulsion droplets^41^. In the present study, the amount of zeta potential was in the medium level, therefore it could lead to particle stability and preventing agglomeration.

#### Moisture content and AW of microcapsules

As shown in Table 1, the moisture content of prepared microcapsules ranged from 4.38 ± 3.50 to 8.56 ± 2.22%, indicating a favorable drying process. Moisture content affects the chemical and mechanical properties and storage stability (agglomeration) of microcapsules^42^. Our results showed that moisture content decreased with increasing Arabic gum and sodium alginate concentration. The high levels of Arabic gum and sodium alginate improved emulsion stability and surface tension and increased emulsion viscosity and caused moisture decrease. Arepally and Goswami (2019) showed the increasing of Arabic gum and maltodextrin concentration led to the moisture content reduction of capsules that these findings were similar to our results. The moisture content of the prepared probiotic microcapsules in various studies differed and was dependent on the type of coating materials and drying method^43^. In all microcapsules, the amount of AW was less than 0.2. In general, microcapsules with AW < 0.1–0.2 are stable from microbiological and biochemical aspects^40^. When AW is lower than 0.1, the probiotic death rate was increased due to oxidation of the membrane^44^. In a study carried out by Fazilah et al.^45^, AW level of microcapsules based on Arabic gum was lower than 0.3, which is approximately similar to our findings.

#### The survival of free and encapsulated *L. plantarum* during sequential incubation in simulated gastrointestinal conditions

**Figure 2:**
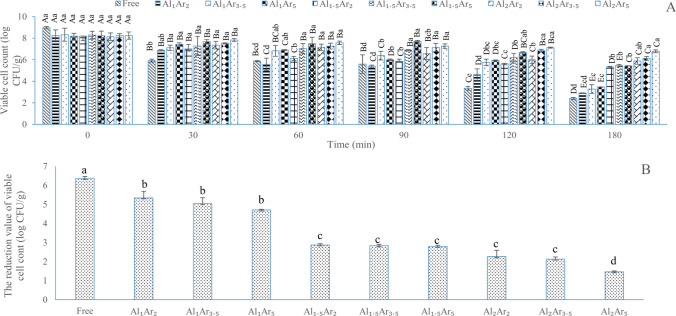
(**A**) The survival (log CFU/g) of free and microencapsulated *L. plantarum* after incubation in simulated gastric juices for 0, 30, 60, 90, 120 and 180 min and sequentially in stimulated intestinal juice containing 0.6% bile salt solutions at 37 °C for 180 min. B: The total reduction value of the number of viable cells of *L. plantarum* after incubation in simulated gastric juices (180 min) and sequentially in stimulated intestinal juice (180 min) (**B**) Values are expressed as mean ± standard deviation (*n* = 3). In (**A**), Lowercase letters above columns indicate significant difference (*P* ≤ 0.05) among different treatments for the same storage period and uppercase letters above columns indicate significant difference (P ≤ 0.05) among different storage period for each treatment. In (**B**), lowercase letters above columns indicate significant difference (*P* ≤ 0.05) among different treatments. Free indicates free (non-microencapsulated) *L. plantarum*; and AlAr shows *L. plantarum* microencapsulated with levels of 1, 1.5 and 2% sodium alginate (Al) and 2, 3.5 and 5% Arabic gum (Ar).

The microencapsulation of *L. plantarum* within sodium alginate and Arabic gum had a significant effect (P ≤ 0.05) on its survivals increment during exposure to simulated gastrointestinal juice**.** By increasing incubation time of *L. plantarum* during exposure to simulated gastrointestinal juice, a continuous reduction in number of probiotic cells was observed (Fig. 2A). The highest (6.37 ± 0.10 log CFU/g) and lowest (1.46 ± 0.05 log CFU/g) of reduction were related to free *L. plantarum* and *L. plantarum* microencapsulated by 2% sodium alginate and 5% Arabic gum, respectively (Fig. 2B). The higher concentration of sodium alginate and Arabic gum resulted in thicker double-layer structure that could have greater protective impacts against the violent environmental factors such as simulated gastrointestinal conditions, therefore it could increase probiotic stability and viability^46^.

#### The survival of *L. plantarum* during jam storage in different conditions

**Figure 3:**
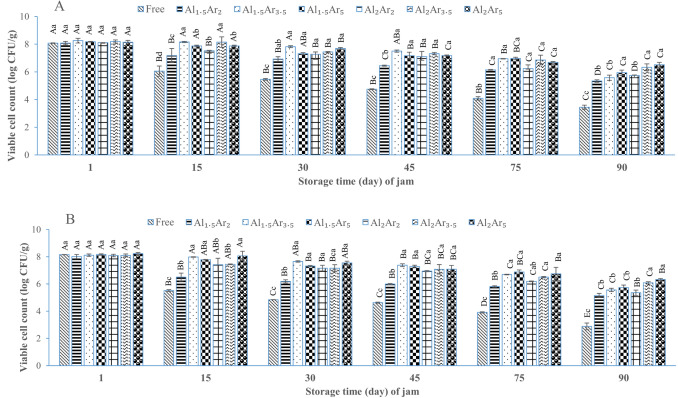
Changes in free and microencapsulated *L. plantarum* count (log CFU/g) during probiotic rose petal jam storage at 4 °C (**A**) and 25 °C (**B**). The total reduction value of the number of viable cells of *L. plantarum* after 90 days of storage at 4 °C (**C**) and 25 °C (**D**). Values are expressed as mean ± standard deviation (*n* = 3). In (**A**) and (**B**), lowercase letters above columns indicate significant difference (*P* ≤ 0.05) among different treatments for the same storage period and uppercase letters above columns indicate significant difference (*P* ≤ 0.05) among different storage period for each treatment. In (**C**) and (**D**), lowercase letters above columns indicate significant difference (*P* ≤ 0.05) among different treatments. Free indicates free (non-microencapsulated) *L. plantarum;* and AlAr shows *L. plantarum* microencapsulated with levels of 1.5 and 2% sodium alginate (Al) and 3.5 and 5% Arabic gum (Ar).

The results of *L. plantarum* survival during jam storage under different conditions are shown in Fig. 3. In all treatments, the count of *L. plantarum* decreased over time (Fig. 3A and B). The survival of free *L. plantarum* was much lower than the microencapsulated form. The low pH and AW of jam lead to probiotic death^17,47^. However, microencapsulation protects probiotic against the mentioned conditions. The viability of *L. plantarum* in jam samples stored at room temperature was significantly (*P* ≤ 0.05) lower than those stored at refrigerator temperature. The high storage temperature causes the increment of cell metabolism and death of probiotic^48^. The reduction level of free *L. plantarum* after 90 days of storage at refrigerator and room temperature was 4.63 ± 0.08 and 5.27 ± 0.35 log CFU/g, respectively (Fig. 3C and D). In jam samples containing *L. plantarum* microencapsulated with 2% sodium alginate and 3.5% or 5% Arabic gum, stored for 90 days at room or refrigerator temperature, the bacterial count was higher than the acceptable level (10^6^ CFU/g).

The count of free *L. plantarum* in jam stored for 30 days at refrigerator temperature was less than 10^6^ CFU/g. Our finding was similar to the study performed by Randazzo et al.^10^, where, the count of free *L. ramensus* in peach jam was less than 10^6^ CFU/g after 30 days of storage at refrigerator temperature.

As results show, the utilization of sodium alginate and Arabic gum for microencapsulation of *L. plantarum* could increase the viability of this bacteria in jam. Therefore, it is a suitable strategy for enhancing the viability of *L. plantarum* inoculated rose petal jam.

### Effect of storage time and temperature on the physicochemical, textural and sensorial characteristics of probiotic and non-probiotic rose petal jam

#### pH and acidity

**Figure 4:**
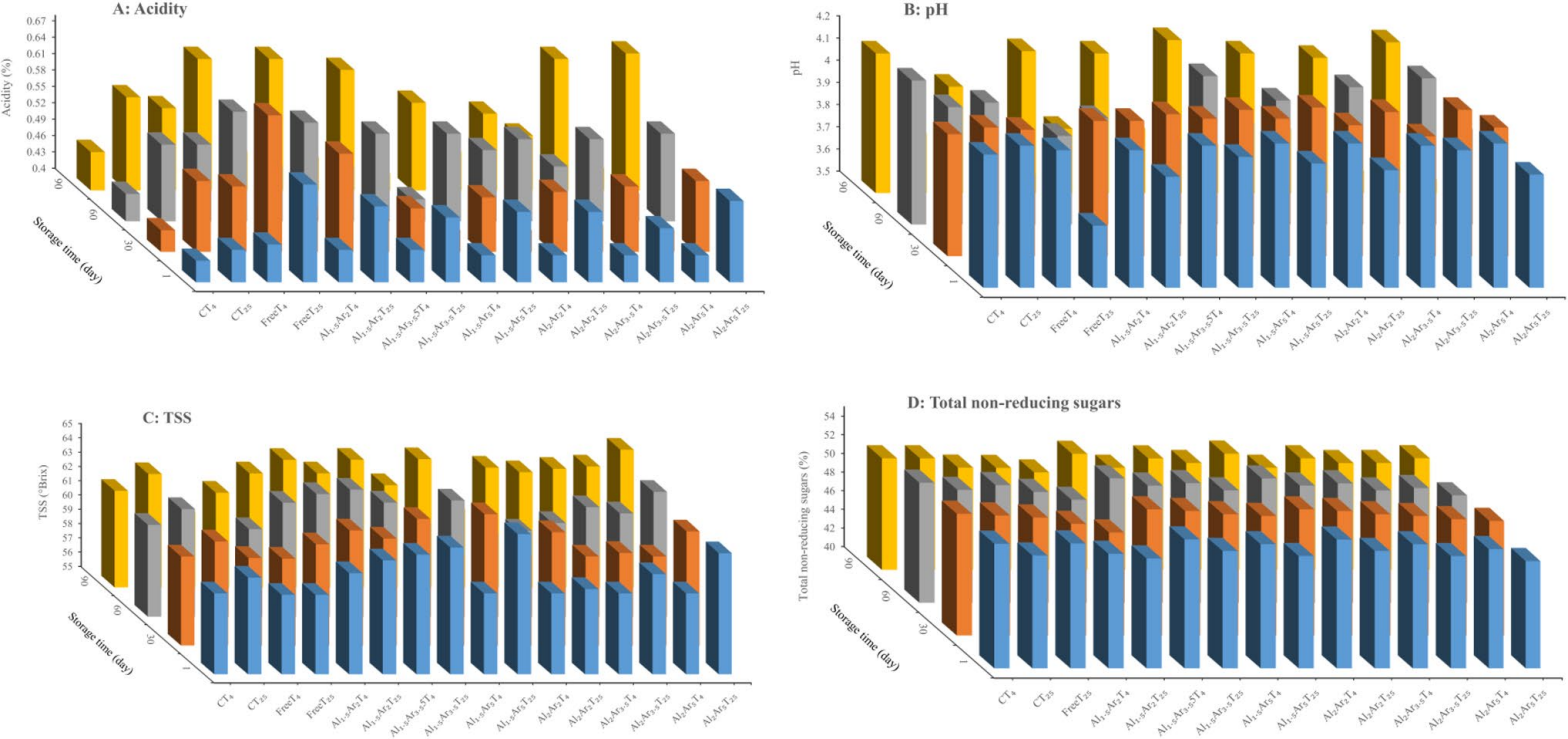
Changes in chemical properties of control (non-probiotic) and probiotic rose petal jams during storage in 4 °C and 25 °C. (**A**) Acidity; (**B**) pH; (**C**) Total soluble solids (TSS); (**D**) Total non-reducing sugar. C indicates control (non-probiotic) jam; Free indicates probiotic jam containing free (non-microencapsulated) *L. plantarum;* and AlAr shows probiotic jam containing *L. plantarum* microencapsulated with levels of 1.5 and 2% sodium alginate (Al) and 3.5 and 5% Arabic gum (Ar). T_4_ and T_25_ indicate storage time of 4 and 25 °C, respectively.

The acidity of the produced jams ranged from 0.44–0.65% (in citric acid) and the pH value ranged from 3.44 to 4.15 (Fig. 4A and B). In all the samples, pH significantly decreased during the storage period, while the acidity significantly increased (*p* ≤ 0.05). These findings were similar to the results reported in previous studies^49,50^. There was no significant difference (*P* > 0.05) between the pH values and acidity percentages of the non-probiotic and probiotic jams stored for a given time. Therefore, the addition of free and encapsulated *L. plantarum* into rose petal jam has no effect on its pH and acidity. In contrast to our results, previous studies have reported that inoculation of probiotics into products, such as cake and juice, could change the pH and the acidity percentage during the storage period^51,52^. The increment of acidity during probiotic jam storage was related to dissociation of organic acids over the time^49^.

#### TSS

In all probiotic and non-probiotic jam samples, the amount of TSS increased during the storage period (Fig. 4C), although this increment was not significant (*P* > 0.05). There was no significant difference (*P* > 0.05) between TSS of the different treatments at a given temperature and time (Fig. 4C). During the storage period, due to conversion of insoluble constitutes to soluble substances, the amount of TSS increased^53^.

#### Total non-reducing sugars

The amount of non-reducing sugar in the jam samples decreased during the storage period (Fig. 4D). There was no significant (*P* > 0.05) difference in the levels of non-reducing sugar of the probiotic and non-probiotic jam samples (*P* > 0.05). During the storage period, sucrose is hydrolyzed to glucose and fructose due to increased acidity^10^. The amount of non-reducing sugar that decreased during the storage period was also found in other jams, such as apricot jam and coconut jam^53,54^, which is in agreement with the result of our study.

#### Color

The addition of the free and microencapsulated form of *L. plantarum* did not have a significant effect (*P* > 0.05) on the L^*^, a^*^, and b^*^ color parameters of the rose petal jam (Table 2). However, the brightness (L^*^ value) of the non-probiotic and probiotic rose petal jam samples decreased significantly during the storage period (*P* ≤ 0.05). There was no significant difference (*P* > 0.05) in the a^*^ and b^*^ color values of the samples stored at room temperature or those stored in the refrigerator, although their color values increased during the 90-day storage period. The reduction of the L^*^ value and the increment of the a^*^ and b^*^ color values during the storage period indicate that the sample darkened due to the formation of brown pigments as a result of the non-enzyme browning reaction^55^. Previous studies have reported that probiotics did not change the color of the product^52^.

**Table 2:** Changes in color (L^∗^, a^∗^, and b^∗^) of plain and probiotic rose petal jams during storage in 4 °C and 25 °C.

Parameter	storage temperature (°C)	Storage time (day)	Treatment							
			C	Free	Al_1.5_Ar_2_	Al_1.5_Ar_3.5_	Al_1.5_Ar_5_	Al_2_Ar_2_	Al_2_Ar_3.5_	Al_2_Ar_5_
L*	4	1	50.50 ± 1.73^Aa^	49.75 ± 2.21^Aa^	49.23 ± 0.89^Aa^	51.33 ± 1.53^Aa^	47.50 ± 10.47^Aa^	45.25 ± 3.30^Aab^	49.25 ± 0.96^Aa^	52.25 ± 0.95^Aa^
	25		49.50 ± 1.30^BAa^	52.00 ± 4.76^Aa^	52.36 ± 2.00^Aa^	48.00 ± 7.74^Aa^	49.50 ± 3.69^Aa^	49.75 ± 1.70^Aa^	47.75 ± 3.09^Aa^	51.75 ± 0.96^Aa^
	4	45	42.33 ± 9.61^Ab^	43.33 ± 7.23^Ab^	44.12 ± 0.98^Aab^	44.67 ± 5.86^Aab^	45.67 ± 0.58^Aab^	42.33 ± 5.03^Aab^	46.00 ± 3.47^Aa^	45.00 ± 1.73^Aab^
	25		45.00 ± 3.00^Aab^	41.00 ± 4.35^Ab^	45.78 ± 1.02^Aab^	41.75 ± 13.22^Ab^	46.00 ± 4.35^Aab^	45.67 ± 1.71^Aab^	43.33 ± 9.86^Aab^	46.00 ± 2.00^Aab^
	4	90	41.25 ± 2.87^Ab^	38.50 ± 2.88^Aac^	42.56 ± 3.45^Ab^	41.00 ± 16.79^Ab^	43.75 ± 5.25^Aab^	40.75 ± 1.50^Ab^	39.25 ± 1.50^Ab^	42.25 ± 4.99^Ab^
	25		40.25 ± 6.60^Ab^	37.00 ± 1.82^Ac^	40.87 ± 5.68^Ab^	39.00 ± 1.82^Ab^	42.25 ± 6.13^Ab^	40.50 ± 5.00^Ab^	35.25 ± 3.20^Ab^	42.50 ± 7.76^Ab^
a*	4	1	37.50 ± 4.12^Ac^	42.00 ± 3.74^Aab^	36.45 ± 1.25^Ac^	37.00 ± 6.68^Ab^	36.50 ± 5.25^Ab^	37.75 ± 7.13^Ac^	38.00 ± 1.15^b^	38.33 ± 1.53^Ac^
	25		39.50 ± 1.29^bAc^	40.75 ± 0.96^Ab^	37.05 ± 1.98^Ac^	37.75 ± 6.13^Ab^	42.25 ± 1.89^Aa^	42.00 ± 2.16^Ab^	37.50 ± 1.91^Ab^	41.75 ± 2.98^Ab^
	4	45	42.67 ± 7.57^Ab^	41.67 ± 4.93^Aab^	38.10 ± 2.06^Ac^	42.25 ± 3.86^bA^	43.75 ± 4.11^Aa^	37.67 ± 1.15^Ac^	42.67 ± 2.08^Aa^	42.75 ± 3.77^Ab^
	25		42.00 ± 2.94^Ab^	39.67 ± 1.53^Ab^	41.23 ± 3.61^b^	49.67 ± 1.53^Aa^	43.33 ± 5.13^a^	45.33 ± 2.08^Aa^	41.67 ± 5.78^Aa^	43.33 ± 4.51^Ab^
	4	90	44.50 ± 2.89^Ab^	43.50 ± 2.64^Aa^	46.25 ± 1.08^Aa^	50.00 ± 2.65^Aa^	44.33 ± 0.58^Aa^	46.50 ± 1.29^Aa^	43.75 ± 4.99^Aa^	44.00 ± 0.00^Ab^
	25		49.33 ± 3.06^Aa^	46.00 ± 3.16^Aa^	49.12 ± 2.35^Aa^	48.75 ± 3.40^Aa^	46.25 ± 2.50^Aa^	47.75 ± 4.11^Aa^	46.50 ± 1.73^Aa^	49.25 ± 3.59^Aa^
b*	4	1	42.00 ± 1.63^Aa^	41.00 ± 4.04^Aa^	38.02 ± 3.05^Ab^	37.00 ± 7.35^Ab^	36.25 ± 5.56^Ab^	42.75 ± 3.50^Ab^	38.25 ± 1.70^Ab^	41.00 ± 1.41^Ab^
	25		41.00 ± 0.81^Aa^	44.00 ± 2.70^Aa^	38.25 ± 4.15^ABb^	38.75 ± 9.56^ABb^	35.50 ± 4.51^Bb^	45.00 ± 4.08^Aab^	40.75 ± 3.40^Ab^	44.50 ± 2.08^Ab^
	4	45	44.00 ± 7.94^ba^	43.67 ± 7.57^Aa^	41.32 ± 2.56^Aa^	41.00 ± 5.20^Ab^	45.33 ± 0.58^Aa^	45.67 ± 3.51^Aab^	45.33 ± 3.79^Aa^	44.67 ± 1.15^Ab^
	25		43.00 ± 4.00^Aa^	45.67 ± 3.21^Aa^	42.65 ± 3.26^Aa^	42.33 ± 2.08^Ab^	45.67 ± 6.43^Aa^	47.00 ± 3.00^Aa^	42.00 ± 7.81^Aab^	46.00 ± 3.60^Aab^
	4	90	46.50 ± 6.19^Aa^	45.75 ± 2.75^Aa^	43.19 ± 5.86^Aa^	48.00 ± 9.41^Aa^	47.50 ± 6.65^Aa^	50.50 ± 2.08^Aa^	49.00 ± 5.09^Aa^	49.50 ± 5.06^Aa^
	25		45.50 ± 3.70^Aa^	45.50 ± 1.29^Aa^	42.78 ± 2.48^Aa^	51.50 ± 3.10^Aa^	47.25 ± 7.13^Aa^	51.50 ± 5.19^Aa^	47.50 ± 3.31^Aa^	50.50 ± 8.50^Aa^

Values are expressed as mean ± standard deviation (n = 3). Means followed by different uppercase letters differ statistically within the same row (*P* ≤ 0.05). Means followed by different lowercase letters differ statistically within the same column (*P* ≤ 0.05). C indicates control (non-probiotic) jam; Free indicates probiotic jam containing free (non-microencapsulated) *L. plantarum;* and AlAr shows probiotic jam containing *L. plantarum* microencapsulated with levels of 1.5 and 2% sodium alginate (Al) and 3.5 and 5% Arabic gum (Ar).

#### Viscosity

The results indicated that the viscosity of all the samples was significantly increased during the storage period (*P* ≤ 0.05). The samples containing microencapsulated *L. plantarum* had a higher viscosity than the samples containing the free *L. plantarum* or those containing non-probiotics (Table 3). The viscosity was impacted by the storage conditions. The samples stored in the refrigerator had greater viscosity than those stored at room temperature. The increased pectin depolymerisation in high temperature results in viscosity reduction^56^. The viscosity of jam depends on various factors, such as pH, TSS and the concentration of sugar, and pectin^57^. In this study, samples containing greater concentration of sodium alginate and Arabic gum had higher viscosity. It seemed that the absorption of water by sodium alginate and Arabic gum during the storage period increases jam samples viscosity.

**Table 3:** Changes in viscosity and texture properties of plain and probiotic rose petal jams during storage in 4 °C and 25 °C.

Parameter	Storage temperature (°C)	Storage time (day)	Treatment							
			C	Free	Al_1.5_Ar_2_	Al_1.5_Ar_3.5_	Al_1.5_Ar_5_	Al_2_Ar_2_	Al_2_Ar_3.5_	Al_2_Ar_5_
Viscosity (centipoise)	4	1	507.81 ± 45.23^Bd^	512.45 ± 71.23^Bd^	617.9 ± 50.18^Ad^	623.93 ± 63.5^Ad^	638.76 ± 65.15^Ad^	655.87 ± 72.72^Ad^	663.84 ± 96.26^Ad^	670.83 ± 87.63^Ad^
	25		437.5 ± 23.23^Be^	445.23 ± 58.4^Be^	553.88 ± 47.93^Ae^	552.95 ± 60.98^Ae^	566.25 ± 61.56^Ae^	581.26 ± 69.58^Ae^	587.53 ± 93^Ae^	593.01 ± 84.35^Ae^
	4	45	751.39 ± 56.45^Bb^	765.24 ± 87.59^Bb^	872.02 ± 61.27^ABb^	870.04 ± 73.48^ABb^	890.97 ± 74.44^Ab^	914.59 ± 83.68^Ab^	930.09 ± 107.57^Ab^	943.68 ± 99.30^Ab^
	25		564.93 ± 72.43^Bc^	578.35 ± 88.22^Bc^	688.16 ± 53.87^Ac^	681.71 ± 66.25^Ac^	698.23 ± 66.28^Ac^	706.15 ± 74.9^Ac^	715.67 ± 97.42^Ac^	723.89 ± 89.91^Ac^
	4	90	851.39 ± 71.34^Ba^	881.45 ± 87.59^Ba^	989.9 ± 67.6^ABa^	971.2 ± 77.69^Ba^	994.96 ± 76.93^ABa^	1006.03 ± 87.62^Aa^	1025.35 ± 110.12^Aa^	1042.04 ± 103.61^Aa^
	25		733.21 ± 76.24^Bb^	745.23 ± 73.64^Bb^	845.23 ± 60.01^ABb^	851.66 ± 72.07^Ab^	871.95 ± 74.52^Ab^	882.18 ± 82.32^Ab^	897.03 ± 104.9^Ab^	909.85 ± 97.88^Ab^
Firmness (N)	4	1	4.85 ± 0.31^Bb^	4.89 ± 0.61^Bb^	4.93 ± 0.37^Bab^	4.96 ± 0.5^Bab^	5.08 ± 0.51^Ba^	5.23 ± 0.53^Bb^	5.30 ± 0.79^Bb^	6.62 ± 0.88^Ac^
	25		4.28 ± 0.12^Bb^	4.34 ± 0.49^Bc^	4.43 ± 0.36^Bb^	4.40 ± 0.49^Bb^	4.5 ± 0.48^Bb^	4.64 ± 0.51^Bc^	4.69 ± 0.76^Bc^	5.88 ± 0.84^Ad^
	4	45	5.63 ± 0.27^Ba^	5.75 ± 0.67^Ba^	5.82 ± 0.37^Ba^	5.73 ± 0.49^Ba^	5.86 ± 0.47^Ba^	5.96 ± 0.48^Ba^	6.06 ± 0.74^Ba^	9.25 ± 0.99^Ab^
	25		4.39 ± 0.43^Bb^	4.49 ± 0.7^Bc^	4.61 ± 0.32^Bb^	4.49 ± 0.44^Bb^	4.63 ± 0.41^Bb^	4.68 ± 0.42^Bc^	4.74 ± 0.67^Bc^	7.02 ± 0.92^Ac^
	4	90	5.59 ± 0.36^Ba^	5.85 ± 0.61^Ba^	6.01 ± 0.37^Ba^	5.83 ± 0.47^Ba^	5.93 ± 0.42^Ba^	6.05 ± 0.44^Ba^	6.19 ± 0.69^Ba^	10.14 ± 1.04^Aa^
	25		4.95 ± 0.47^Bb^	5.07 ± 0.5^Bb^	5.05 ± 0.31^Bb^	5.03 ± 0.42^Ba^	5.19 ± 0.42^Ba^	5.23 ± 0.4^Bb^	5.33 ± 0.66^Bb^	8.79 ± 0.98^Ab^
Cohesiveness (N)	4	1	0.53 ± 0.04^Aa^	0.53 ± 0.06^Aa^	0.54 ± 0.04^Aa^	0.54 ± 0.06^Aa^	0.55 ± 0.06^Aa^	0.57 ± 0.06^Aa^	0.59 ± 0.09^Aa^	0.66 ± 0.09^Aa^
	25		0.49 ± 0.02^Aa^	0.49 ± 0.05^Aa^	0.50 ± 0.04^Aa^	0.5 ± 0.05^Aa^	0.51 ± 0.05^Aa^	0.53 ± 0.06^Aa^	0.56 ± 0.09^Aa^	0.59 ± 0.08^Aa^
	4	45	0.61 ± 0.03^Aa^	0.62 ± 0.07^Aa^	0.63 ± 0.04^Aa^	0.62 ± 0.05^Aa^	0.63 ± 0.05^Aa^	0.64 ± 0.05^Aa^	0.69 ± 0.08^Aa^	0.63 ± 0.1^Aa^
	25		0.50 ± 0.05^Aa^	0.51 ± 0.07^Aa^	0.52 ± 0.04^Aa^	0.51 ± 0.05^Aa^	0.52 ± 0.05^Aa^	0.53 ± 0.05^Aa^	0.58 ± 0.08^Aa^	0.7 ± 0.09^Aa^
	4	90	0.61 ± 0.04^Aa^	0.64 ± 0.06^Aa^	0.65 ± 0.04^Aa^	0.63 ± 0.05^Aa^	0.64 ± 0.05^Aa^	0.66 ± 0.05^Aa^	0.70 ± 0.08^Aa^	0.98 ± 0.1^Aa^
	25		0.56 ± 0.05^Aa^	0.58 ± 0.05^Aa^	0.57 ± 0.04^Aa^	0.57 ± 0.05^Aa^	0.59 ± 0.05^Aa^	0.59 ± 0.05^Aa^	0.65 ± 0.08^Aa^	0.88 ± 0.1^Aa^
Chewiness (N)	4	1	0.73 ± 0.05^Aa^	0.72 ± 0.08^Aa^	0.74 ± 0.06^Aa^	0.74 ± 0.08^Aa^	0.75 ± 0.08^Aa^	0.78 ± 0.08^Aa^	0.81 ± 0.13^Aa^	0.85 ± 0.12^Ab^
	25		0.73 ± 0.03^Aa^	0.74 ± 0.08^Aa^	0.75 ± 0.06^Aa^	0.75 ± 0.07^Aa^	0.76 ± 0.07^Aa^	0.79 ± 0.08^Aa^	0.84 ± 0.12^Aa^	0.83 ± 0.12^Ab^
	4	45	0.81 ± 0.05^Aa^	0.82 ± 0.1^Aa^	0.84 ± 0.06^Aa^	0.82 ± 0.07^Aa^	0.84 ± 0.07^Aa^	0.85 ± 0.07^Aa^	0.92 ± 0.11^Aa^	0.94 ± 0.14^Aa^
	25		0.74 ± 0.07^Aa^	0.77 ± 0.09^Aa^	0.78 ± 0.06^Aa^	0.76 ± 0.07^Aa^	0.78 ± 0.07^Aa^	0.79 ± 0.07^Aa^	0.86 ± 0.11^Aa^	0.89 ± 0.13^Aa^
	4	90	0.83 ± 0.06^Aa^	0.87 ± 0.08^Aa^	0.88 ± 0.06^Aa^	0.86 ± 0.07^Aa^	0.88 ± 0.07^Aa^	0.9 ± 0.07^Aa^	0.96 ± 0.11^Aa^	0.98 ± 0.14^Aa^
	25		0.80 ± 0.07^Aa^	0.82 ± 0.07^Aa^	0.81 ± 0.06^Aa^	0.81 ± 0.07^Aa^	0.84 ± 0.07^Aa^	0.84 ± 0.07^Aa^	0.92 ± 0.11^Aa^	0.96 ± 0.14^Aa^

Values are expressed as mean ± standard deviation (n = 3). Means followed by different uppercase letters differ statistically within the same row (*P* ≤ 0.05). Means followed by different lowercase letters differ statistically within the same column (*P* ≤ 0.05). C indicates control (non-probiotic) jam; Free indicates probiotic jam containing free (non-microencapsulated) *L. plantarum;* and AlAr shows probiotic jam containing *L. plantarum* microencapsulated with levels of 1.5 and 2% sodium alginate (Al) and 3.5 and 5% Arabic gum (Ar). N: Newton.

#### Textural properties

The firmness of the jam samples was dependent on the coating of *L. plantarum*, the total solids content, the storage period and temperature (Table 3). The highest amount of firmness (10.14 N) was found in the jam sample containing *L. plantarum* microencapsulated by 2% sodium alginate and 5% Arabic gum; there was no significant difference (*P* > 0.05) in the firmness value of the other samples. The firmness value was increased during the storage period, and the samples stored in the refrigerator had a higher firmness value than those stored at room temperature. Our results were in contrast with those reported by Rababah et al. (2014), who found no significant differences in the firmness value of cherry jam stored at 25 °C, 35 °C, and 45 °C for 15 days^58^. The previous studies demonstrated that hydrocolloid compounds, including Arabic gum and sodium alginate caused firmer texture in the jam samples^59,60^.

No significant difference (*P* > 0.05) was observed in the chewiness and cohesiveness of the various samples. In a study done by Teixeira et al*.* (2020), the results showed that the addition of orange peel in orange jam resulted in decreased adhesiveness and increased chewiness^61^. Moreover, Younis et al*.* (2015) showed that the addition of sweet lemon peel in jam caused firmness and chewiness increment and adhesiveness reduction^62^.

#### Sensory properties

**Figure 5:**
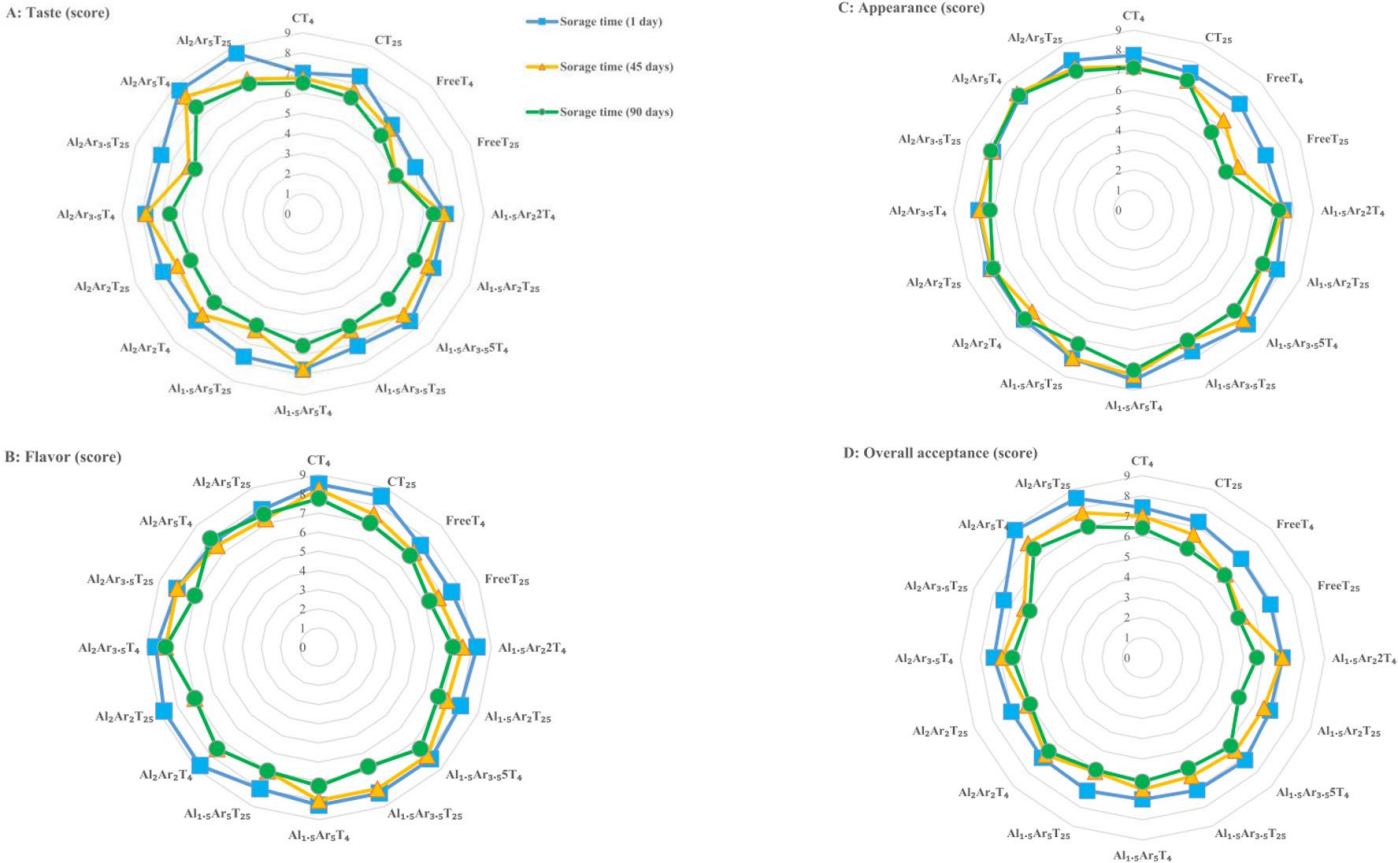
The score of sensory properties of non-probiotic and probiotic rose petal jams. (**A**) Taste; (**B**) Flavor; (**C**) Appearance; (**D**) Overall acceptance. C indicates control (non-probiotic) jam; Free indicates probiotic jam containing free (non-microencapsulated) *L. plantarum;* and AlAr shows probiotic jam containing *L. plantarum* microencapsulated with levels of 1.5 and 2% sodium alginate (Al) and 3.5 and 5% Arabic gum (Ar). T_4_ and T_25_ indicate storage time of 4 and 25 °C, respectively.

The sensory properties (Fig. 5) of the jam samples depended on the storage time, the temperature conditions, and the type of inoculated probiotic bacteria (free or microencapsulated form) that was used. The score for the sensory properties of the jam samples, including taste, flavor, appearance, and overall acceptability, showed a decreasing trend during the storage period, which is similar to the results reported in a previous study^53^. The reduction of the sensory property score of the jam samples that were stored at room temperature and contained free probiotic bacteria was significantly greater than the scores of the samples that were stored in the refrigerator that contained microencapsulated probiotics (*P* ≤ 0.05). The anthocyanin destruction and the Millard reaction that occurred during jam storage could result in a decrease in the sensory score. Furthermore, the rate of these reactions increases at a higher temperature^54^. In general, the increment of sodium alginate and Arabic gum concentration, improved the sensory score so that the highest score for taste and overall acceptance was related to the jam sample containing *L. plantarum* microencapsulated by 2% sodium alginate and 5% Arabic gum so that this sample had significant difference (*P* ≤ 0.05) with other samples; thus, it was chosen as the most acceptable jam. There was no significant difference (*P* > 0.05) in the flavor and appearance score among the non-probiotic and probiotic jams. Sodium alginate and Arabic gum improve the texture of jam; therefore, they had a positive impact on the sensory score. Previous studies indicated that the sensory properties of various products containing microencapsulated probiotics were similar to or higher than the products containing free probiotics^51,52,63,64^.

## Conclusion

This study was the first attempt to produce probiotic rose petal jam. The microencapsulation of *L. plantarum* was performed with sodium alginate and Arabic gum to increase its viability during jam storage. There are no considerable differences between the physicochemical properties, cohesiveness and chewiness of non-probiotic jam and probiotic jam. However, the sensory assessment indicated that the jam sample containing microencapsulated *L. plantarum* with 2% sodium alginate and 5% Arabic gum had a higher taste and overall acceptance score than the other samples. Therefore, in general, it could be said that microencapsulation of *L. plantarum* with 2% sodium alginate and 5% Arabic gum and its inoculation into rose petal jam resulted in a probiotic product manufactured with physicochemical and textural properties that are similar to non-probiotic jam, and it had better sensory attributes. After 90 days of storage, the probiotic jam under room temperature and refrigerator conditions produced samples that contained a viable count of *L. plantarum* in an acceptable level (> 10^6^ CFU/g).

## Supplementary Information

Supplementary Information 1.

Supplementary Information 2.

Supplementary Information 3.
