# Supplementary material for: The assessment of microencapsulated Lactobacillus plantarum survivability in rose petal jam and the changes in physicochemical, textural and sensorial characteristics of the product during storage
Source: Sci Rep. 2022 Apr 13;12:6200. doi: 10.1038/s41598-022-10224-w (PMC9007973; doi:10.1038/s41598-022-10224-w)
Supplement: Supplementary file 1 — Supplementary Information 1. [file 41598_2022_10224_MOESM1_ESM.docx]

^
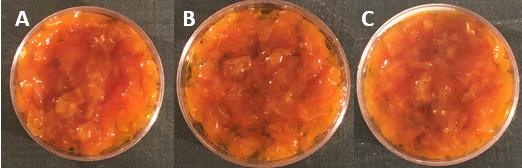
^

Supplementary Figure S1. Photos of non- probiotic rose petal jam (A), rose petal jam containing free *L. plantarum* (B) rose petal jam containing microencapsulated *L. Plantarum*
