# Supplementary material for: The assessment of microencapsulated Lactobacillus plantarum survivability in rose petal jam and the changes in physicochemical, textural and sensorial characteristics of the product during storage
Source: Sci Rep. 2022 Apr 13;12:6200. doi: 10.1038/s41598-022-10224-w (PMC9007973; doi:10.1038/s41598-022-10224-w)
Supplement: Supplementary file 2 — Supplementary Information 2. [file 41598_2022_10224_MOESM2_ESM.docx]

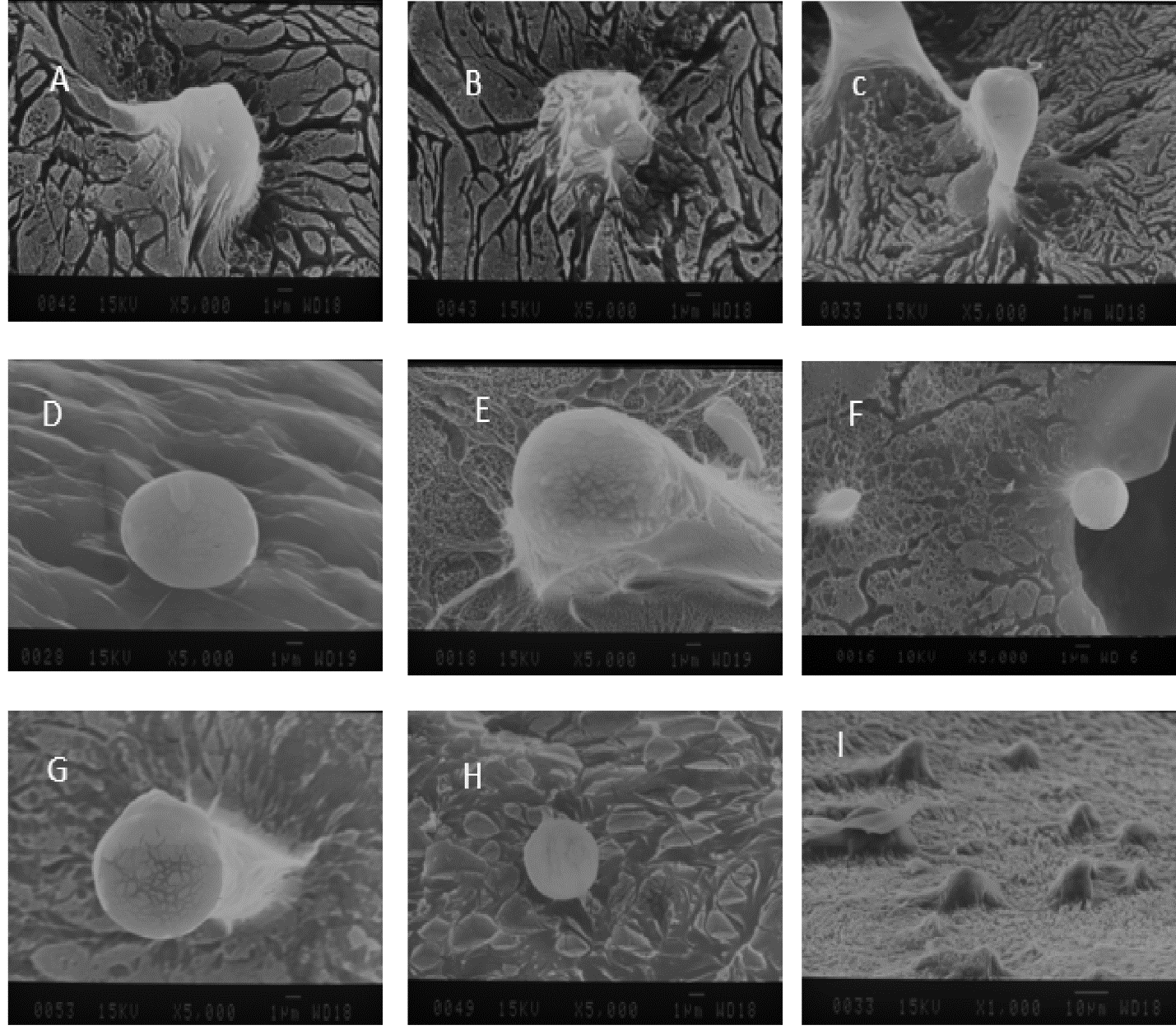


Supplementary Figure S2. SEM images (with a 5000× magnification and 1 µm resolution) of *L. plantarum* microencapsulated with sodium alginate (Al) in various levels (1, 1.5 and 2%) and Arabic gum (Ar) in different levels (2, 3.5 and 5%).

A: A_l_1Ar_2_, B:Al_1_Ar_3.5_, C:Al_1_Ar_5_, D:Al_1_._5_Ar_2_ E:Al_1.5_Ar_3.5_,F:Al_1.5_Ar_5_, G:Al_2_Ar2, H:Al_2_Ar_3.5_ and I: Al_2_Ar_5_
